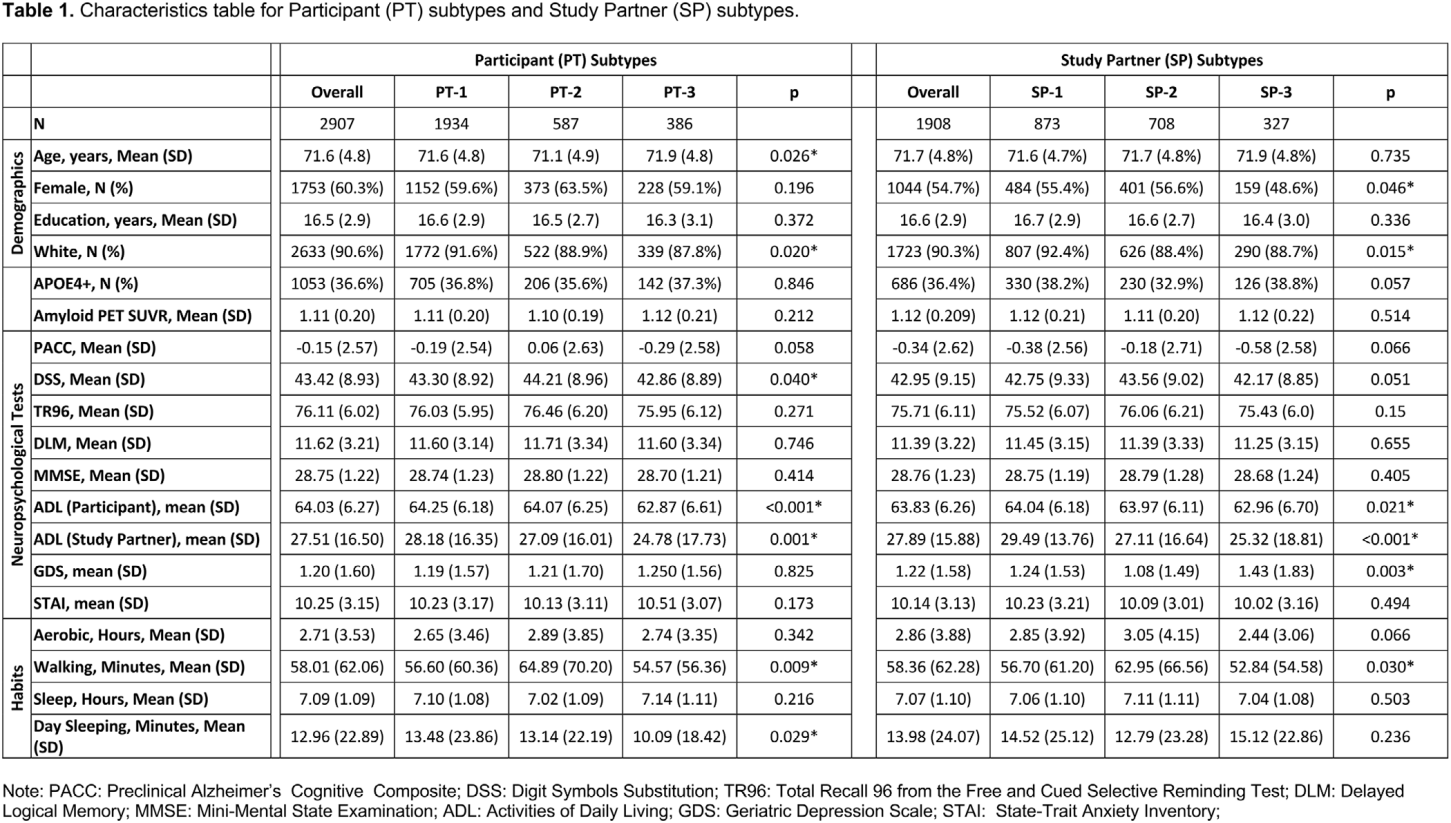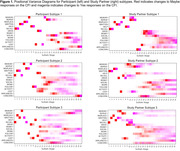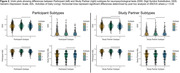# Investigating heterogeneity of cognitive and functional decline using Subtype and Stage Inference machine learning

**DOI:** 10.1002/alz.094316

**Published:** 2025-01-09

**Authors:** Kellen K. Petersen, Bhargav Teja Nallapu, Idris Demirsoy, Richard B. Lipton, Ellen Grober, Laura Rabin, Elham Ghanbarian, Ali Ezzati

**Affiliations:** ^1^ Albert Einstein College of Medicine, Bronx, NY USA; ^2^ University of California, Irvine, Irvine, CA USA; ^3^ Department of Neurology, Albert Einstein College of Medicine, Bronx, NY USA; ^4^ Brooklyn College of the City University of New York, Brooklyn, NY USA

## Abstract

**Background:**

Alzheimer’s disease (AD) is a heterogeneous disease with different clinical phenotypes and pathophysiological subtypes. Identifying cognitive/functional subtypes in AD could elucidate the diverse clinical progression patterns. The Cognitive Function Index (CFI), a 15‐item questionnaire completed by participants and study partners, captures aspects of cognitive and functional decline. We investigated participant heterogeneity using the CFI and compared differences between subtypes based on participant or study partner reports.

**Methods:**

Participants were 4486 cognitively unimpaired older adults from the Anti‐Amyloid Treatment in Asymptomatic Alzheimer’s (A4) Study. All participants and study partners completed the CFI. Participants had baseline demographics, neuropsychological test scores, and amyloid PET measures available. The Staging and Subtype Inference (SuStaIn) algorithm was independently applied to participant (PT) and study partner (SP) CFI reports to identify subtypes. Differences between subtypes were assessed using Analysis for Variance (ANOVA) with post hoc analysis.

**Results:**

Table 1 summarizes participant characteristics. Analysis of PT and SP CFI responses independently identified three subtypes with distinct progression patterns. Notably, 35.2% and 57.5% of participants showed no progression based on PT and SP reports, respectively. All subtypes, except SP‐2, had CFI items related to difficulties with recall, needing written reminders, and misplacing items as the first items of suspected decline (Figure 1). Clusters PT‐1 (66.5%) and SP‐1 (45.8%) were the subtypes with most frequent progression pattern (Figure 1). Subtypes PT‐2 and SP‐2 had the highest scores on Digit Symbol Substitution test, walked the most, and for SP‐2 lowest Geriatric Depression Scores (p<0.05 for all; Figure 3). Subtypes PT‐3 and SP‐3 scored lowest on the participant Activities of Daily Living (ADL; p<0.05). Additionally, PT‐3 scored lowest on the Study Partner ADL.

**Conclusion:**

We identified distinct subtypes from both participant and study partner CFI reports. Post hoc analysis indicated consistency within subtypes identified from the two sources of information. Subjective reports on cognitive decline could serve as a valuable tool for classifying participants into different risk groups.